# Can computational efficiency alone drive the evolution of modularity in neural networks?

**DOI:** 10.1038/srep31982

**Published:** 2016-08-30

**Authors:** Colin R. Tosh

**Affiliations:** 1 School of Biology, Newcastle University, Ridley Building 2, Newcastle upon Tyne NE1 7RU, UK

## Abstract

Some biologists have abandoned the idea that computational efficiency in processing multipart tasks or input sets alone drives the evolution of modularity in biological networks. A recent study confirmed that small modular (neural) networks are relatively computationally-inefficient but *large* modular networks are slightly more efficient than non-modular ones. The present study determines whether these efficiency advantages with network size can drive the evolution of modularity in networks whose connective architecture can evolve. The answer is no, but the reason why is interesting. All simulations (run in a wide variety of parameter states) involving gradualistic connective evolution end in non-modular local attractors. Thus while a high performance modular attractor exists, such regions cannot be reached by gradualistic evolution. Non-gradualistic evolutionary simulations in which multi-modularity is obtained through duplication of existing architecture appear viable. Fundamentally, this study indicates that computational efficiency alone does not drive the evolution of modularity, even in large biological networks, but it may still be a viable mechanism when networks evolve by non-gradualistic means.

Modularity in a network of interactions occurs when the network is subdivided into relatively autonomous, internally highly connected components^1^. Modularity is found in diverse systems ranging in scale from an animal’s body organ system and the brain, to protein and other molecular networks. When undertaking a multipart task, or processing a multipart input stream, it seems intuitively reasonable that a modular network might work better because different modules can process different parts of the task or different parts of the input (a ‘task’ in this context is the series of operations a network must perform on inputs to produce outputs that maximise fitness). A number of studies using the neural network model formulation (which has also been used to model other non-neural systems such as gene regulatory networks^2^) have demonstrated, however, that modularity is not necessarily the most computationally efficient system property under a broad range of network parameter states for such tasks (see ref. 3, the ‘network parameter state’ is the value of the set of model parameters that determine the behaviour of the network). The most obvious reason why any trait might evolve, then, (because it is efficient at an apparently relevant task) would not appear to apply to modularity. This lead to a number of studies investigating other selective factors that may have promoted its near ubiquitous presence in nature: node connection costs, synaptic weight noise, modularly varying network goals and overcoming the problem of ‘catastrophic forgetting’^4^,^5^,^6^,^7^. Another study using a more explicitly gene regulatory network formulation showed that specialisation to conflicting tasks is a plausible mechanism^8^. Studies using an evolutionary genetic framework have related correlated stabilising selection and the existence of epistasis^9^, and varying pleiotropy and correlated directional selection^10^, with the evolution of modularity. Numerous other explanations have been proposed for the evolution of modularity including several non-adaptive mechanisms^1^.

Most previous work on the evolution of modularity has used model networks that are quite small, usually with 50 nodes or less. However, as Tosh and McNally^11^ pointed out, even relatively small scale biological networks can consist of thousands or millions of nodes (neurons, proteins etc.). There are good reasons to believe that the performance of modular networks relative to non-modular ones should increase with networks size. An influential idea^12^ beginning with^13^ indicates that modularity may evolve in networks to reduce pleiotropic effects among network regions serving different functions. We might expect this problem to be especially severe in large, non-modular networks, where irrelevant inputs to regions processing relevant information for a given task may be vast. Feed-forward neural networks (the models used in this article) are known to be inefficient in some aspects of performance when they are too big or small relative to the number of training examples. Specifically, very large networks are known to become over capable in that they can implement numerous solutions that are consistent with the training data, but most of these are poor approximations to the actual problem^14^ (but see ref. 15). Introducing modularity may simply reduce the number of weights in the network and so reduce one aspect of the effective size of the network.

Tosh and McNally^11^ set about assessing the importance of size (gauged as node number in networks with the same, constant, number of nodes in each network layer) on the relative computational efficiency (the relative fitness) of network modularity by constructing ‘small’ (25 nodes) and larger (145 node–still relatively small by biological standards but computationally manageable with today’s computer hardware) neural networks of one fixed modular connective architecture and two non-modular architectures (Fig. 1). These networks undertook the task of differentiating a set of randomly selected inputs from other inputs, and modules processed a different part of each input, integrating information late in the computational process to decide response at each processing iteration (Fig. 1). Such an arrangement has analogies in numerous real biological information processing systems^16^,^17^,^18^. In agreement with previous studies the small modular network was less computationally efficient than one of the non-modular architectures, but the large modular network was significantly more efficient than both of the non-modular ones (Fig. 1). In other words, the relative benefits of modularity increased with network size. The authors considered robustness of this effect with regard to system parameter state and found it robust to almost all of the parameters considered: in large networks modularity is at least as good and often a more efficient architecture than non-modular networks of the same size. Thus in networks of the large size and form considered by the authors modularity should represent a significant attractor in state space for networks that are allowed to evolve their connective architecture in time. It is interesting to note that modularity of bacterial metabolic networks increases with network size with most of this increase occurring between networks of approximately 50 to 200 nodes^19^.

This article considers whether large connectively evolving networks initially parameterised and undertaking the same task as Tosh and McNally^11^ (with system characteristics subsequently subject to an extensive sensitivity analysis) do indeed evolve towards modularity through advantages in computational efficiency alone or whether alternative solutions are found. I find that they do not evolve modularity but principally because state space contains one or more potent local attractors and the gradualistic algorithm (run in a wide range of parameter combinations) cannot reach regions in which the advantages of modularity may be realised. Additional simulations indicate that a non-gradualistic, duplicative evolutionary mechanism presents a viable route to multi-modularity through performance advantage alone.

Biologists have long been interested in the relative contribution of mutations of small and large phenotypic effect to evolutionary change^20^,^21^. Early proponents of the view that large mutations contribute significantly to evolution were discredited because of the extremity of their position^20^,^22^, but with the rise of evolutionary developmental biology^23^ this issue is being reconsidered. While changes in gene regulatory sequences need not result in large phenotypic changes, it is this type of mutation that we might expect to result in substantial changes. Clarke and Arthur^24^ suggest that the assertion^25^ that mutations of small effect make a predominant, but not exclusive, contribution to evolutionary change is probably correct. Similarly Hoekstra and Coyne^26^ conclude that “Adaptation and speciation probably proceed through a combination of *cis*-regulatory and structural mutations, with a substantial contribution of the latter”. Nevertheless, there are numerous examples where mutation in gene regulatory sequences leading to substantial phenotypic change appear to have contributed to evolution^23^.

This debate is central to considerations of how evolution traverses fitness surfaces to reach fitness optima. Kauffman and colleagues^27^,^28^ argue that fitness surfaces of complex networked systems will commonly contain many troughs that evolution will have difficulty escaping from on its way to global optima. Kauffman’s solution was to demonstrate that expression states of genomic systems are not continuous but undergo discrete shifts^27^. The originator of the evolutionary landscape metaphor, Sewall Wright, also appreciated the problem of local maxima and proposed that it is overcome by a process and population subdivision and genetic drift; the Shifting Balance Theory^29^. Other researchers have suggested this issue of the local maximum in evolution is an imaginary one, consequent of our tendency to visualise evolution in too few dimensions. When evolution is envisaged multidimensionally, fitness surfaces may be more realistically seen as ‘holey’^30^. Many computer scientists and computational biologists working on complex networks are aware of the problem of local maxima during system optimization^31^,^32^.

In summary then, this study finds that modularity will not evolve through computational advantage alone but this may be down to the problem of gradualist evolution being prey to potent non-modular local attractors. Evolution by a non-gradualistic replicative mechanism may provide an alternative route to modularity. Alternatively or additionally (as indicated by studies cited above), factors other than computational efficiency may contribute to the evolution of modularity.

## Methods

The starting point for simulations was the large (96 input nodes, 48 hidden nodes, 1 output node) network of state 1 from^11^ and here I describe this network. Modifications to this basic form are described subsequently.

### Starting network state and commonalities of all networks

**Figure 1:**
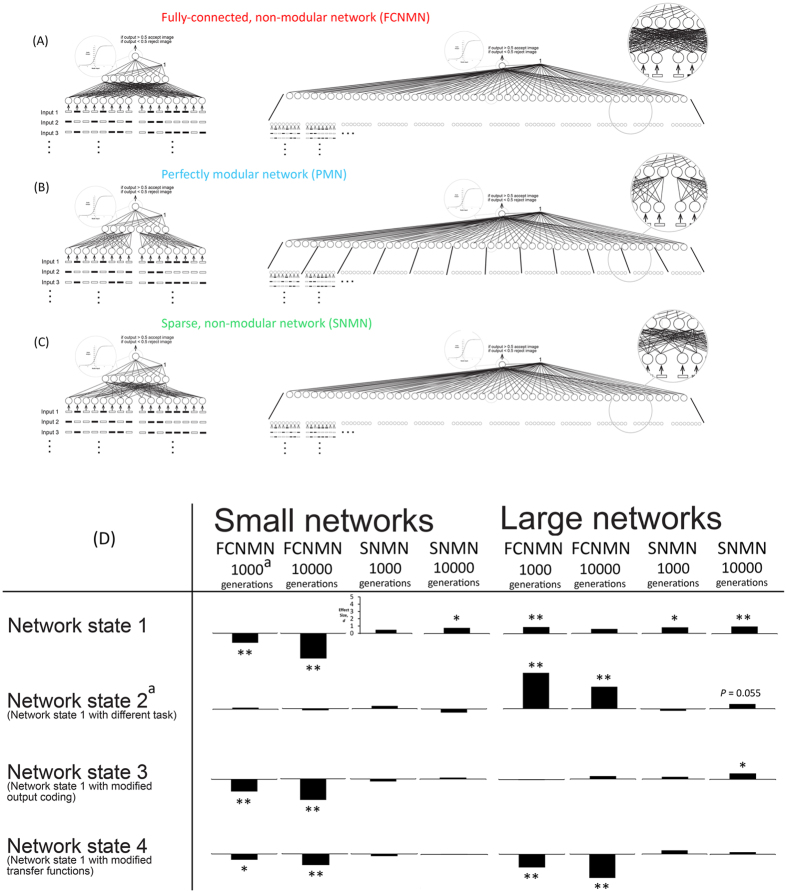
A summary of the methods and results of ref.^11^. Parts **(A–C)** show small and large instances of the modular (blue) and non-modular (red and green) networks. Part (**D)** shows the size of effects of non-modular network structure (relative to the modular network) on fitness of networks at 1000 and 10000 generations of training. Bars pointing upwards indicate that the modular PMN network performs better than the non-modular network and those pointing down indicate the opposite. Significant effects (gauged using the *t*-test) are marked with asterisks: *indicates 0.05 < *P* < 0.01, **indicates *P* < 0.01 and absence of an asterix indicates *P* > 0.05. Networks are studied in four states through modification of network characteristics to determine the robustness of effects.

**Figure 2:**
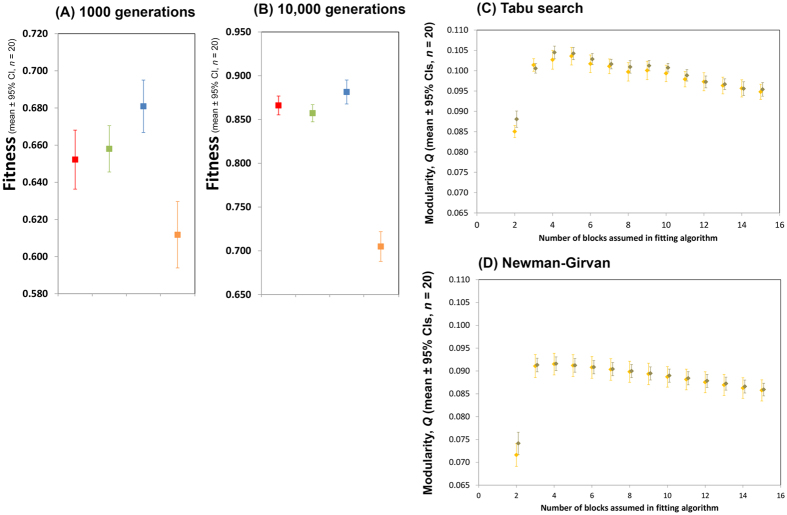
Evolution of modularity in the ‘starting network state’ described in the Methods section. Parts (**A,B)**, red, green and blue show the performance at 1000 and 10,000 generations of the fully connected non modular network (red), the sparse non-modular network (green), and the perfectly modular network (blue) of ref. 11, where networks were conformed as present but had a variety of fixed connective architectures and only weights were allowed to evolve. The data in yellow is the performance of the networks in the starting state described in the methods section where both weights and connective architecture have been allowed to evolve. Parts (**C,D)** show the final level of evolved modularity between the input and hidden layer in the structurally evolving networks after 10,000 generations. Modularity has been assed using a Tabu search and the Newman-Girvan algorithm. The grey points are a control in which the degree distribution of each node in the input layer of the networks at the end of the evolutionary simulation has been randomized. No difference between grey and yellow bars means essentially that there is no more evolved modularity than would be expected at random. An additional analysis in which goodness of fit of cliques designated by the Tabu algorithm are assessed using a Hamming fit is shown in Figure S11. Results are essentially as here.

I used 3-layer, feed-forward artificial neural networks with McCulloch-Pitts neurons in the hidden and output layers^33^, activation functions of the form: output = 1/(1 + (*e* ^ -β*input)), *β* = 1, and trainable bias. Input nodes simply received the numerical value of inputs and had weighted connections to the hidden layer. Hidden and the output nodes had trainable bias. The connective architecture of networks used at the commencement of connective evolution was the large FCNMN (fully connected non-modular network) shown in Figs 1 and 2. In FCNMNs each node in the input layer had a weighted connection to each node in the hidden layer. There was also a weighted connection between each node of the hidden layer and the single output node. Each input set into a network was a 256 × 8 × 12 array with each 256 × 8 sub-element consisting of all binary combinations of eight array elements, positionally randomised with respect to row. For each network architecture/network size combination, 20 replicates of 50 networks (the latter is the population size in the genetic training algorithm) were initiated by choosing each connection weight from a uniform random distribution between −3 and 3. Each of the 20 reps of the network was allocated a randomly selected subset of 100 inputs from the relevant input sets and it was the task of the networks within the genetic algorithm to maximize output activity (output >0.5) in response to these while minimising response (output <0.5) in response to the remaining 156 inputs. Formally, fitness was defined as


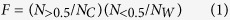


where *N*_*>0.*5_ is the number of output responses >0.5 to the predesignated input set to which networks should respond with high output activity, *N*_*C*_ is the size of the predesignated input set to which networks should respond with high output activity (here 100), *N*_*<0.*5_ is the number of correct responses i.e. output <0.5 to the remaining set of inputs, *N*_*W*_ is the size of the remaining set of inputs (here 156). This gave a metric with a value of 1 when behaviour was perfect and 0 when completely imperfect and some level of performance at both types of task (acceptance and rejection of appropriate subsets) was required for fitness >0. Note that while each of the 20 reps was allocated a unique size-100 input set, the same set of 20 was used between network architecture types within network size classes. The genetic training algorithm proceeded by selecting the top performing 10 nets from the 50 in each generation, cloning each of these networks five times, and mutating each weight within these by adding a number selected from a random normal distribution with mean 0 and SD 0.25 to form the next generation.

### Connective evolution

As well as networks undergoing weight evolution in active connections, I allowed a connection between two nodes of a network to become active or inactive. Connections between the hidden and output layer always remained active but now during each generation of training any active connection between input and hidden nodes could become inactive. Similarly any inactive connection could come active. This transition to opposite state occurred with a probability of 0.001 at each connection within each generation of training. During the period in which a connection was inactive, its weight value continued to mutate as described above. One may imagine a biologically analogous scenario where the genes responsible for a biological structure are ‘switched off’ but may continue to mutate neutrally. Modifications to this mutational regime, namely allowing only unidirectional mutation from ‘on’ to ‘off’ are described in subsequent sections along with other modifications to evolutionary simulations. The motivation behind the various modifications described below will be apparent as simulation outputs are described in the results section.

### Repetitions of simulations with modified network and evolutionary algorithm characteristics

#### A small network

The task to be performed was as the starting network state (to respond with high activity to the same randomly chosen set of 100 inputs, see Methods section ‘*Starting network state and commonalities of all networks’*) but the size of the network (and number of data input sets) was decreased. The input layer now contained 16 nodes in the input layer relative to 96 in the starting network state, and 8 nodes in the hidden layer relative to 48 in the starting network state. The output layer remained one node.

#### A lower rate of weight mutation

The larger starting network size (see Methods section ‘*Starting network state and commonalities of all networks’*) was used and the ‘severity’ of the weight mutation regime was decreased. Each weight now mutated with a probability of 1/1000 and when mutation did occur the number to be added to the weight was chosen from a random normal distribution with mean 0 and SD 1.

#### Initiation of connective evolution from a complete absence of connections between the input and hidden layer

The larger starting network size was used (see Methods section ‘*Starting network state and commonalities of all networks’*) and the lower rate of weight mutation described above (see Methods section ‘*A lower rate of weight mutation’*) was maintained. Additionally connective evolution was started from a complete absence of connections between the input and hidden layers of the network. This contrasts to previous simulations in which simulations began from a fully connected network.

#### Irreversible connective loss

The larger starting network size (see Methods section ‘*Starting network state and commonalities of all networks’*) was used and the lower rate of weight mutation described above was maintained (see Methods section ‘*A lower rate of weight mutation’*). In the starting network state and previous simulations a connection between layers 1 and 2 may be lost but can potentially return later in the simulation after substantial drift. Here networks were initiated from full connectivity between layers 1 and 2 and mutated at the same rate as previously but lost connections could now no longer return.

#### A different network task

While fundamentally the ‘task’ of the network was changed here this necessarily involved changes to multiple parts of the system and this is desirable as it tests the robustness of effects to major state change. The same input set as previous simulations was employed (see Methods section ‘*Starting network state and commonalities of all networks’*) but now all 1s within 140 rows of the 256 row input set were converted to a number from a random uniform distribution between 0 and 0.5, with each row receiving a different random number. This procedure was repeated 20 times to produce inputs for the 20 replicates. If one wishes to apply a visual analogy to the input-receiver system we have modelled, the 116 inputs that are unmodified are visually ‘intense’ whereas the modified inputs are visually ‘dull’. The task of the network was to respond with high output activity (output >0.5) to the ‘dull’ inputs and to respond with low activity (output <0.5) to the ‘intense’ inputs. Formally, fitness was defined as (the number of correct responses i.e. output >0.5 to the size-140 input subset)/140 * (the number of correct responses i.e. output <0.5 to the remaining 116 inputs)/116. Some aspects of this new input-task system (numbers of inputs in the ‘dull’ subset, decision to ‘accept’ rather than reject dull inputs) are, within bounds, arbitrary and were chosen in some cases simply to ensure the system differed substantially from the starting network state. The larger starting network size was used (see Methods section ‘*Starting network state and commonalities of all networks’*) and the lower rate of weight mutation described above (1/1000, see Methods section ‘*A lower rate of weight mutation’*) was maintained, and networks were started from full connectivity between layers 1 and 2. I repeated simulations using the fixed, perfectly modular connective architecture of^11^. Here only weights evolved.

#### A very large network

All aspects of the simulation (except the lower rate of weight mutation described in the Methods section ‘*A lower rate of weight mutation’* above-1/1000-which was maintained) were as the starting network state (see Methods section ‘*Starting network state and commonalities of all networks’*) but the size of network (and number of data input sets) was increased. The input layer now contained 144 nodes in the input layer relative to 96 in the starting network state, and 72 nodes in the hidden layer relative to 48 in the starting network state. The output layer remained one node.

#### Temporal separation of weight and connective evolution

Starting with fully connected networks, a population of 50 networks was connectively mutated with a probability of assuming an off state if on and vice versa at each connection between the input and hidden layer of 1/400. Each of these networks was then optimized using weight updating only as described in the starting network state above (see Methods section ‘*Starting network state and commonalities of all networks’*) for 400 generations. Weights were updated with a probability of 1/400 and when mutation did occur the number to be added to the weight was chosen from a random normal distribution with mean 0 and SD 1. This whole process of structural mutations and weight adaptation, which temporally separates different mutation processes and superficially mimics structural evolution with within-generation learning, was repeated 400 times. The smaller number of generation run in this simulation, and the lower number of reps (n = 8), was due to the computationally intensive nature of the simulation. Mutation rate was raised slightly to allow a reasonable region of state space to be traversed across a lower number of generations.

#### A more aggressive mutational regime

Networks were returned to the ‘starting network state’ (see Methods section ‘*Starting network state and commonalities of all networks’*) in which all weights mutated in each generation and additionally the rate of connective mutation was increased from 1/1000 to 1/100. This was this most aggressive mutational regime considered in this study.

#### Modular evolution through replication of existing substructure

**Figure 3:**
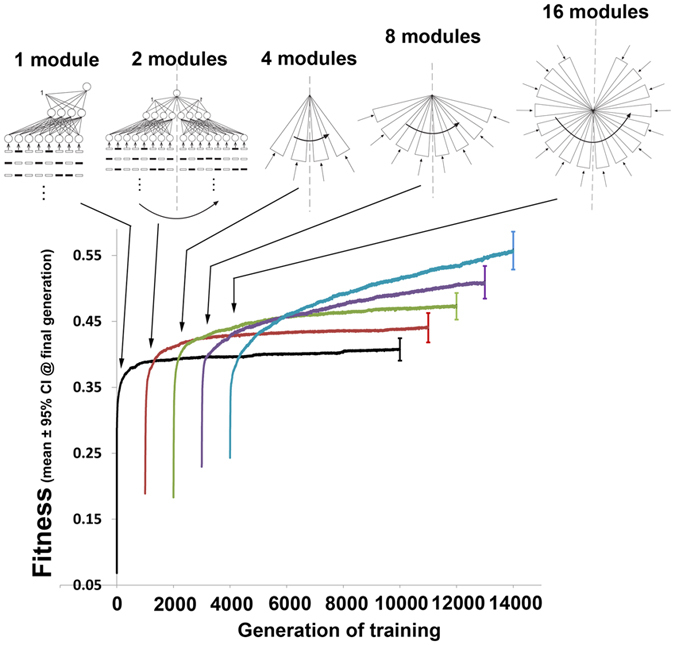
Fitness of networks as modularity is evolved through duplicating of existing subunits. Connection weights evolve gradualistically but after 1000 generations there is a mutation that leads to the replication of all existing architecture and connection weights and a new input stream is acquired by the new architecture. The new network then continues to evolve weights gradualistically from this starting point. The performance of this mutated network can be compared with the performance of the network from which it derived after this large-effect mutation occurs. *n* = 20 for construction of means and CIs.

A standard 8-4-1 fully connected neural network (Fig. 3 ‘1 module’) configured as in the starting network state (Methods section ‘*Starting network state and commonalities of all networks’* above, with the exception of weight mutation rate: each weight mutated with a probability of 1/1000 and when mutation did occur the number to be added to the weight was chosen from a random normal distribution with mean 0 and SD 1) was trained through weight evolution only for 10000 generations. After 1000 generations all architecture including evolved weights was duplicated and a new set of inputs acquired to produce a 2-module network (Fig. 3 ‘2 modules’). This apparatus formed the starting point of a further 10000 generations of weight-only evolution in which the weights of both modules could evolve to optimize task. After 1000 generations of this new evolutionary sequence the network again mutated and both modules were replicated exactly to form a 4-module network which then evolved a further 10000 generations from this starting point, again mutating after 1000 generations to produce an 8-module network. This sequence was repeated to finally produce a 16-module network. Network fitness was plotted throughout these network manipulations to determine if evolution via these gross architectural manipulations is viable.

### Quantifying modularity

The evolutionary simulations under each of the parameter scenarios described above was repeated 20 times (*n* = 20, with one exception, *n* = 8, see above). The symmetric association matrix representing connections between the input and hidden layers of each network after 10,000 generations of evolution was input into the UCINET 6 social network analysis package^34^,^35^. I used the ‘factions’ routine to quantify modularity. It assumes a partition of a binary network of adjacencies into *n* groups and counts the number of missing ties within each group summed with the ties between the groups to give a measure of the extent to which the groups form separate clique like structures. It uses a Tabu search minimization procedure to optimize this measure to find the best fit. I presented fit using the *Q* measurement of modularity^36^ and within the search algorithm used a maximum number of iterations in a search series of 20, a different random number seed to create the initial partition each time the algorithm was run, a reverse movement prohibition time of 15 steps, and 3 random starts. Variation of these parameters within computationally manageable bounds made no fundamental difference to the conclusions of the analysis. Selected analyses were repeated using the Girvan-Newman^37^ routine in UCINET 6 and gave similar results (e.g. Fig. 1 Parts A and B). To create a null model to compare evolved networks with, degree of all input nodes in evolved networks was maintained but distribution of connections from each input node was randomised.

## Results

The starting network state of simulations was the large fully connected network of ref. 11 (and see Fig. 1 and Methods). This network is significantly more task efficient than two non-modular architectures of the same size when weights alone are optimized^11^ (Fig. 1). Connections and weights between the input and hidden layers of this network were allowed to evolve using an evolutionary algorithm with relatively small changes to connective architecture and weight values in each generation (gradualistic evolution). The final evolved state of the network was not modular (Fig. 2, parts C and D). Confidence intervals of levels of modularity of evolved and null model networks using both Tabu and Newman-Girvan algorithms for treatment and control were broadly overlapping. In the case of least overlap (two blocks assumed within each fitting algorithm) treatment networks appeared marginally less modular (more connectively homogenous) than the randomly conformed control networks. The mean fitness value of networks at 1000 and 10,000 generations (by which time fitness had reached and asymptote) was considerably less than that of networks of the same size with fixed connective architecture whose weights were allowed to evolve at the same rate as presently^11^ (Fig. 2, parts A and B). Evolution of the present networks and those of ref. 11 was additionally started from the same area in weight space. Taken together, these observations indicate that the present networks that are allowed to evolve in both weight and connective architecture (presence/absence of a connection) are ending evolution in local attractors. At the end of simulations the number of connection between input and hidden layers was 2306 ± 14.2 (mean ± 95% CI, *n* = 20): 0.50 of the possible connections between the input and hidden layers were realised.

I then underwent a sensitivity analysis to determine if the absence of modularity in the evolved networks was due simply to the parameters and system configuration chosen to run this initial simulation. As this is a substantial analysis and a clear generalization emerges, it is presented in full in the SI with only a textual summary presented here. I evolved a smaller network (16-8-1) with the expectation^11^ that this would not evolve to a modular state and this prediction was upheld (Figure S1). Interestingly, evolved networks realized similar fitness to those whose weights only were evolved^11^, so there was no evidence of a local attractor evolutionary end point. Returning to the original large network, I next reasoned that the absence of modularity in evolved structure could be due to an imbalance of weight and connective mutation regimes. The weight mutation regime chosen followed from^11^: all weights mutated in each generation by a small amount. In contrast only 1/1000 of the connections in each network mutated. Mutation in weight could, therefore, be a more important determinate of fitness than connective evolution and constrain connective evolution. I reduced the rate of weight mutation to 1/1000 but increased the size of each mutation. This made little difference to predictions: evolved networks were still non-modular and ended in a lower performance state than networks of this size can achieve^11^ (Figure S2). I maintained this new weight-connection mutation regime for most of the remainder of the sensitivity analysis (reasoning that it would be less likely to constrain structural evolution) and started evolution off from networks in which there were no connections between the input and hidden layer. This did not result in the evolution of modularity between these layers and the final solution was suboptimal in terms of performance (Figure S3). I return to these simulations later in the results section. I wondered if the continual background drift of weights associated with connections that have disappeared but that later reappear was responsible for the suboptimality of evolved states, so repeated simulations only allowing connections to change their on-off status once. This did not result in the evolution of modularity (networks were less modular than random in fact) and the final solution was suboptimal in terms of performance (Figure S4). I changed the task of the network to be optimized but this did not result in the evolution of modularity and the final solution was suboptimal in terms of performance (Figure S5). I ran even larger networks (144/72/1) with the original task but this did not result in the evolution of modularity (Figure S6). Weight and structural evolution was separated in time during evolution but this did not result in the evolution of modularity (Figure S7). I ran further simulations to demonstrate that while there is not modular connective evolution between the input layers, there is connective evolution of some sort (Figure S8), and simulations to show that modularity is not a transient state during evolution (Figure S9). I lastly considered the possibility that the various mutational regimes I used were not aggressive enough, predisposing evolution to end in local, not global attractor. I returned parameters to the starting network state where all weights evolved in each generation and additionally increased the incidence of connective mutation from 1/1000 to 1/100. This did not result in the evolution of modularity and the final solution was suboptimal in terms of performance (Figure S10). In summary, then, while a previous study^11^ using large structurally static (weight evolving) networks indicated an increase in the relative advantage of modularity with size, the present study with large, fixed-size networks in which connections and weights have been allowed to evolve does not yield significant modularity in any of the simulation states considered. The non-modular evolved endpoints are found to commonly be local attractors as their performance is less that than of the networks of the same size whose weights only were evolved in a previous study^11^.

**Figure 4:**
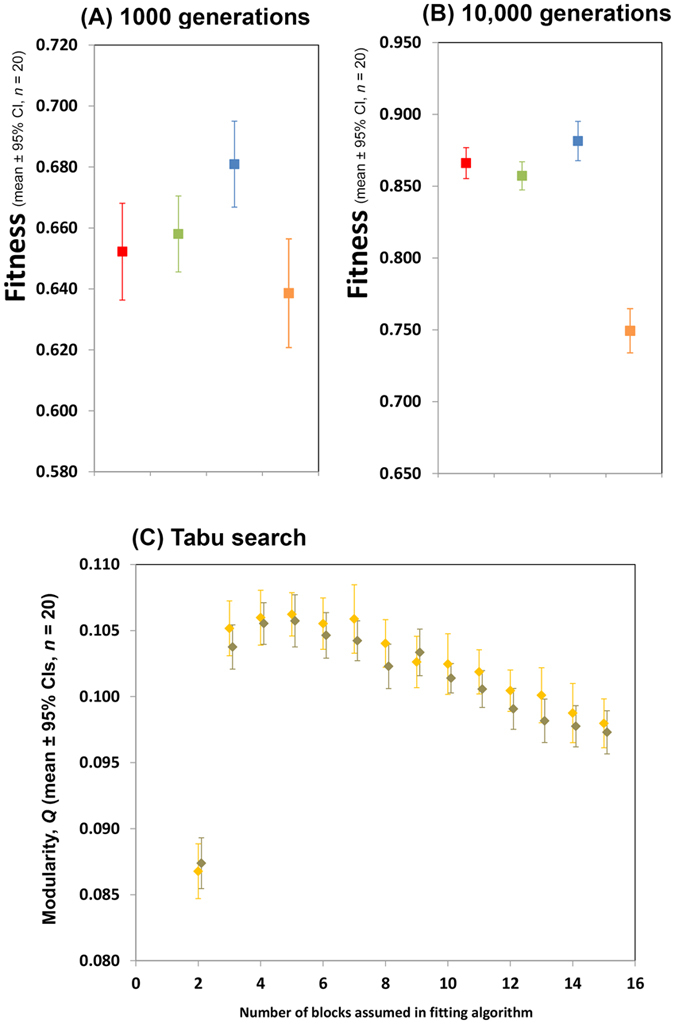
Evolution of modularity with reduced rate of weight mutation in the algorithm in which both networks weights and connections could evolve. Simulations additionally begin from a starting condition in which there are no connections between layers 1 and 2 of the network. Parts (**A,B)**, red, green and blue show the performance at 1000 and 10,000 generations of the fully connected non modular network (red), the sparse non-modular network (green), and the perfectly modular network (blue) of ref. 11, where networks were conformed as present but had a variety of fixed connective architectures and only weights were allowed to evolve. The data in yellow is the performance of the networks where both weights and connective architecture have been allowed to evolve. Part (**C)** shows the final level of evolved modularity between the input and hidden layer in the structurally evolving networks after 10,000 generations. Modularity has been assed using a Tabu search algorithm. The grey points are a control in which the degree distribution of each node in the input layer of the networks at the end of the evolutionary simulation has been randomized. No difference between grey and yellow bars means essentially that there is no more evolved modularity than would be expected at random.

So performance advantage alone does not promote modularity in large fixed size networks but what of networks that may grow? The simulation depicted in Figure S3 (reproduced in Fig. 4) in which networks start from a state with no connections between the input and hidden layers are presented above as simulations with ‘fixed size’ networks. This is, however, a somewhat superficial, pictorial, description as functionally these networks in fact grow gradualistically. Such a network with no connections between the input and hidden layer has no functional parts. After a small number of generations some networks will acquire active connections and with it active nodes. This growth will continue gradualistically until soon all nodes have active connections when growth will continue through the further acquisition of active connections. As indicated above (reproduced in Fig. 4) such a gradualistic growth process does not result in the evolution of modularity in evolving regions. A network of the same functional size (in terms of active nodes) may also be obtained non-gradualistically through replication of existing modular architecture^1^ and I have analysed the fitness trajectory of networks evolving by this means (see *‘Modular evolution through replication of existing substructure’* section of Methods for more details and Fig. 3). There are 3 notable features about the output of these simulations. Firstly, as modules are added the fitness trajectory is generally upwards. Secondly, when new modules are added there is, of course, a period in which fitness of networks is below that of networks that have not undergone the size-increasing mutation. This period is short when networks are small but longer when networks are large, presumably placing an upper limit on the evolution of complexity. Thirdly, evolved weights when duplicated to form a new module are useful when placed in their new context: there is an upwards trend in starting fitness after module duplication. This does not appear to be responsible for the increase in performance of networks with an increasing number of modules as networks with many modules starting off at a similar level of fitness to networks with fewer modules still obtain a higher final fitness level. It could however reduce the duration over which networks perform poorly after module replication, promoting the evolution of modularity.

## Discussion

This article considers whether large connectively evolving networks of the type in Tosh and McNally^11^ do indeed evolve towards modularity through advantages in computational efficiency alone or whether alternative solutions are found. I find, surprisingly, that despite at least one significant attractor for modularity in state space^11^, networks evolving gradualistically always move towards non-modular solutions. On the basis of this result alone this study joins the others reviewed in the introduction indicating that computational efficiency alone is unlikely to be a major driver of the evolution of modularity. The reason for the ubiquitous evolution of non-modularity is, however, unexpected. State space of this network appears to contain one or more potent, local, non-modular attractors. Structural evolution (run in numerous model parameter variants) always ends in states that have significantly lower performance than networks of this size and form can achieve as determined in the previous study^11^. As modularity is favoured in at least one region of state space^11^ but mode of evolution precludes the algorithm reaching such high performance areas I run simulations using an alternative mode in which multi-modularity is evolved through duplication of existing architecture. This is a plausible route by which multi-modularity may evolve through simple computational efficiency advantages. Researchers have identified numerous selective factors that may drive the evolution of modularity (see refs in introduction) thus there is no reason compelling reason to insist that computational efficiency alone may drive the evolution of modularity. Nevertheless, assuming non-gradualistic modes of evolution it still remains a possibility.

I ran evolutionary simulations in a variety of states in an attempt to find a state in which local attractors can be overcome or are less prevalent but was unsuccessful. I can, therefore, state that the large networks considered here are *prone* to evolving towards local attractors but I cannot say with certainty that they *always* evolve towards such attractors or that these attractors cannot be overcome to reach areas of the fitness surface where modularity may be advantageous, as my search was not exhaustive. Relevant to this issue are a number or recent studies showing that complex biological systems may overcome local attractors by evolving their evolvability^12^,^13^,^38^,^39^,^40^,^41^,^42^. Colgrave and Collins^12^ broadly speaking divide evolutionary process that may improve evolvability into those that increase suitable genetic variation within populations and those that influence the structure of the fitness surface by modifying system structure. While I made no conscious attempt to encode evolvability into the present system I may nevertheless have done so because some authors have suggested^13^ that modularity is a form of structural modification that evolves to improve evolvability through the reduction of pleiotropic interactions between component parts of a network. Future work to allow connective and weight mutation characteristics to evolve would increase confidence in my assertion that the local attractors identified here represent genuine barriers to evolutionary progress in the network studied. It is worth mentioning the possibility that end points of structural evolution do not represent local attractors but, rather, fit modular solutions lie so far off in state space from simulation starting points that they cannot be reached in 10,000 generations, but I believe this possibility can be dismissed. Simulations were run in a broad variety of parameter combinations, with variable mutation rates and starting from different points in state space, and a fit modular solution never once evolved. Each network experienced an average of around 5 connective mutations in each generation in the starting state networks. Over 10,000 generations this is sufficient for networks (each with 4608 potential connections between input and hidden layer) to lose and gain all their connections several times over. Lastly, examination of Figures S8 and S9 of the SI indicates that networks reach a non-modular state with stable connection number relatively quickly in evolutionary time. I have been cautious in my use of language, consistently referring to the end point of simulations as ‘local attractors’ which is a general term that can encompass numerous modes of evolutionary attraction. Normally one assumes that simulations ending in local attractors are in local maxima: local multistate fitness ‘hills’ that cannot be escaped without reducing fitness of all individuals in a population. Most likely this is the case in the present simulations but there are other possibilities. For example, I am presently investigating the possibility that connective architecture of gene networks evolves selfishly when mutation rate is high, to nullify the negative impact of mutation. This issue is potentially resolvable by characterising the fitness surface in the region of simulation end points or starting simulations from the high-performance modular attractor of the previous study^11^ but these additional simulations are potentially quite involved, may not produce unambiguous conclusions, and are not directly relevant to the principal aim of this study so they have not been attempted here.

I demonstrated that networks growing through occasional replication of existing architecture display a potentially viable route to multi-modularity. The only barrier to evolution of modularity is a short period of suboptimality immediately after replication with functionality quickly regained in small networks and over a longer period in larger networks. Whether the new, modular evolutionary novelty can survive long enough to produce higher functioning mutants will depend on a number of factors including strength of natural selection and stochasticity within the selection process. It should also be noted that while each of the new modules I have added onto the network carries out a similar task to the others (in order that simulations focus on computational efficiency alone as a driver of modularity; not computational efficiency and the interaction of additional factors), in reality new modules may assume new tasks. This could additionally impact the immediate viability of new modules, potentially promoting establishment of the new module. Another more general point about this study is that it considers fitness of the network as the ultimate determinant of fitness of the possessing organism. In reality fitness of an ‘organ’ such as the network will only be one component of fitness, along with other aspects of behaviour, physiology and organic structure. This is the problem of reductionism. The reason why acquisition of new modules ultimately improves functional efficiency within the system studied may be related to information acquisition^43^. A network may find it difficult to distinguish some ‘good’ from ‘bad’ inputs because these inputs are relatively similar and interactions between different parts of the network may compromise its ability to distinguish them. If the network acquires a new module and a new input stream, the relevant subset of inputs may by chance be more easily distinguishable and the new module can concentrate exclusively on this task with input from the new and original module integrated late during information processing. Mine is not the first study to consider duplication of existing architecture as a potential route to modularity. Sole and Fernandez^44^ demonstrate the emergence of modularity from a network growth process involving architectural duplication followed by connective differentiation within duplicated areas. Perhaps the main insight of the current work is that modular growth through duplicative processes also appears viable from a functional perspective.

The findings of this study are potentially relevant to numerous real biological networks. The networks considered here are characterized by processing different sources of information in relatively independent streams that are later integrated, and such systems are well known in nature: the columnar organisation of the somatic sensory cortex of mammals^16^, the processing of different image attributes within distinct areas of the retina, superior colliculus, lateral geniculate nucleus and early visual cortical areas of primates^17^, and the early visual processing apparatus of insects^18^. If computational efficiency has had a role in driving the evolution of modularity in these systems the present study indicates that this is unlikely to be the case in systems that have evolved gradualistically and this is likely to be reflected in the genetic architecture underlying morphology. It is not clear whether the issue of local attractors during the evolution of networks connective architecture is a relevant issue to previous theoretical studies that have examined the evolution of modularity. Often networks are of a quite different form (reviewed in ref. 3) to the present ones or when they are of the same general form, different input streams are assigned different tasks or quite distinct input sets and the overall task of the network differs from the present networks^467^. Workers in this area may like to revisit their studies to determine if fixed architectures (with evolving weights only) produce higher fitness values than those with evolving weights and connections, as I have done here.

In summary then, multi-modularity is functionally efficient in the large neural networks considered here^1^ but will not evolve gradualistically either through subdivision of a large, connectively homogenous network or through network growth, in part because evolution is prey to pervasive, non-modular local attractors. A viable route to the large multi-modular network is through replication of existing modular architecture. While Wagner *et al.*^1^ in their review of the evolution of modularity term such replicative processes selectively neutral, I additionally show that each replication event (after a brief period of dysfunction) is associated with an upwards trajectory in computational efficiency that will promote the evolution of multi-modularity. It is worth repeating that numerous other selective factors in the evolution of modularity (reviewed in the introduction) are better supported than computational efficiency *per se*, especially in light of the observation here that gradualistic structural evolution does not proceed to modularity under any circumstances. Nevertheless, this study indicates that it could still have a role to play, particularly if is biological networks are evolving by non-gradualistic means.

## Additional Information

**How to cite this article**: Tosh, C. R. Can computational efficiency alone drive the evolution of modularity in neural networks? *Sci. Rep.* **6**, 31982; doi: 10.1038/srep31982 (2016).

## Supplementary Material

Supplementary Information
