# Supplementary material for: Can computational efficiency alone drive the evolution of modularity in neural networks?
Source: Sci Rep. 2016 Aug 30;6:31982. doi: 10.1038/srep31982 (PMC5004152; doi:10.1038/srep31982)
Supplement: Supplementary Information [file srep31982-s1.pdf]

Supplementary information for: Colin R. Tosh 'Can computational efficiency alone drive the evolution of modularity in neural networks?'

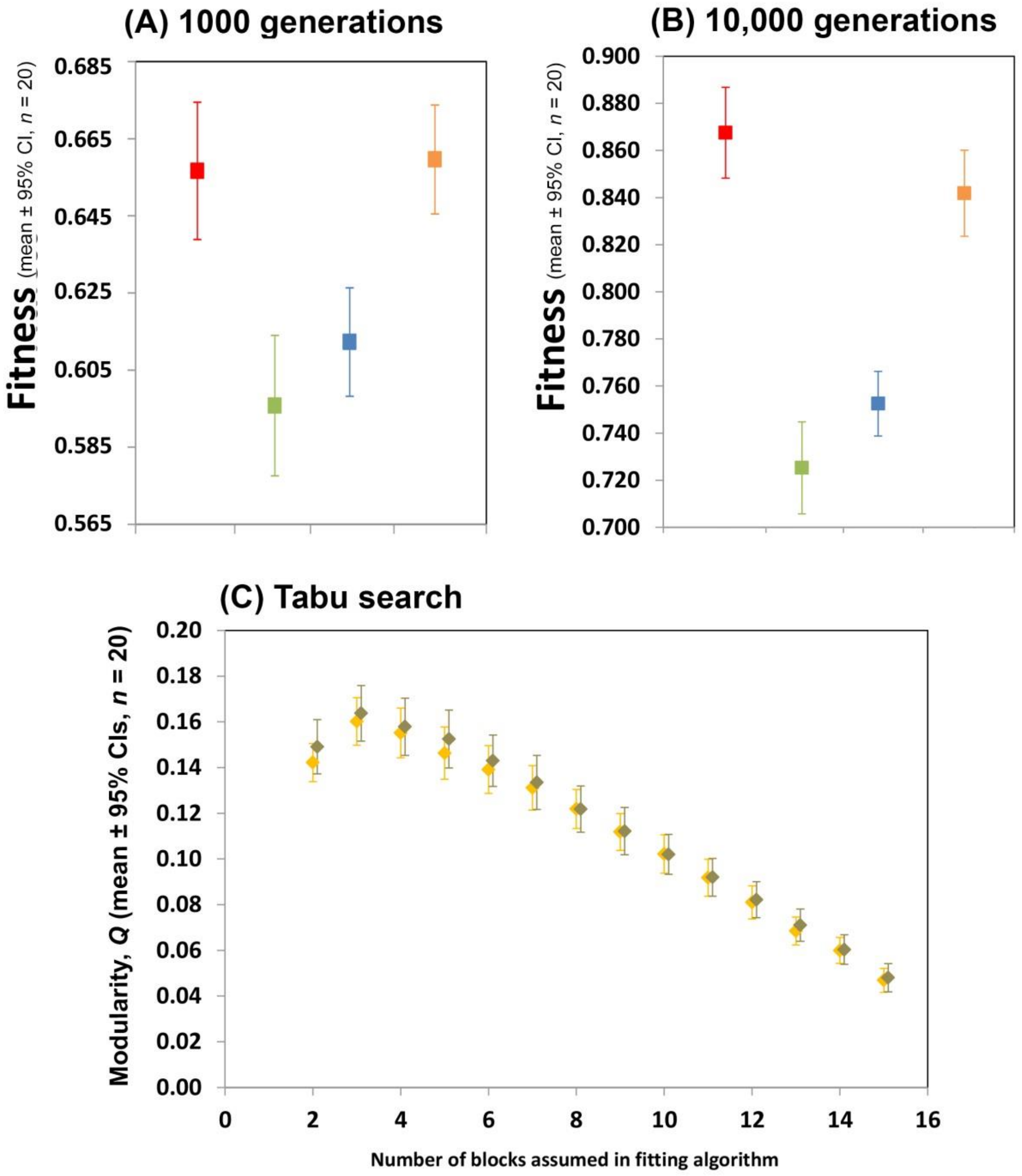

Figure S1. Evolution of modularity in a network with smaller input and hidden layer dimensions than the ‘starting network state’ described in the Methods section. Parts A and B, red, green and blue show the performance at 1000 and 10,000 generations of the (similarly small) fully connected non modular network (red), the sparse non-modular network (green), and the perfectly modular network (blue) of Tosh (2014), where networks were conformed as present but had a variety of fixed connective architectures and only weights were allowed to evolve. The data in yellow is the performance of the networks in the small network where both weights and connective architecture have been allowed to evolve. Part C shows the final level of evolved modularity between the input and hidden layer in the structurally evolving networks after 10,000 generations. Modularity has been assed using a Tabu search algorithm. The grey points are a control in which the degree distribution of each node in the input layer of the networks at the end of the evolutionary simulation has been randomized. No difference between grey and yellow bars means essentially that there is no more evolved modularity than would be expected at random. The number of active connections between input and hidden layer at the end of simulations was 80.4 ± 2.9 (*n* = 20), approximately 0.63 of the possible number of active connections.

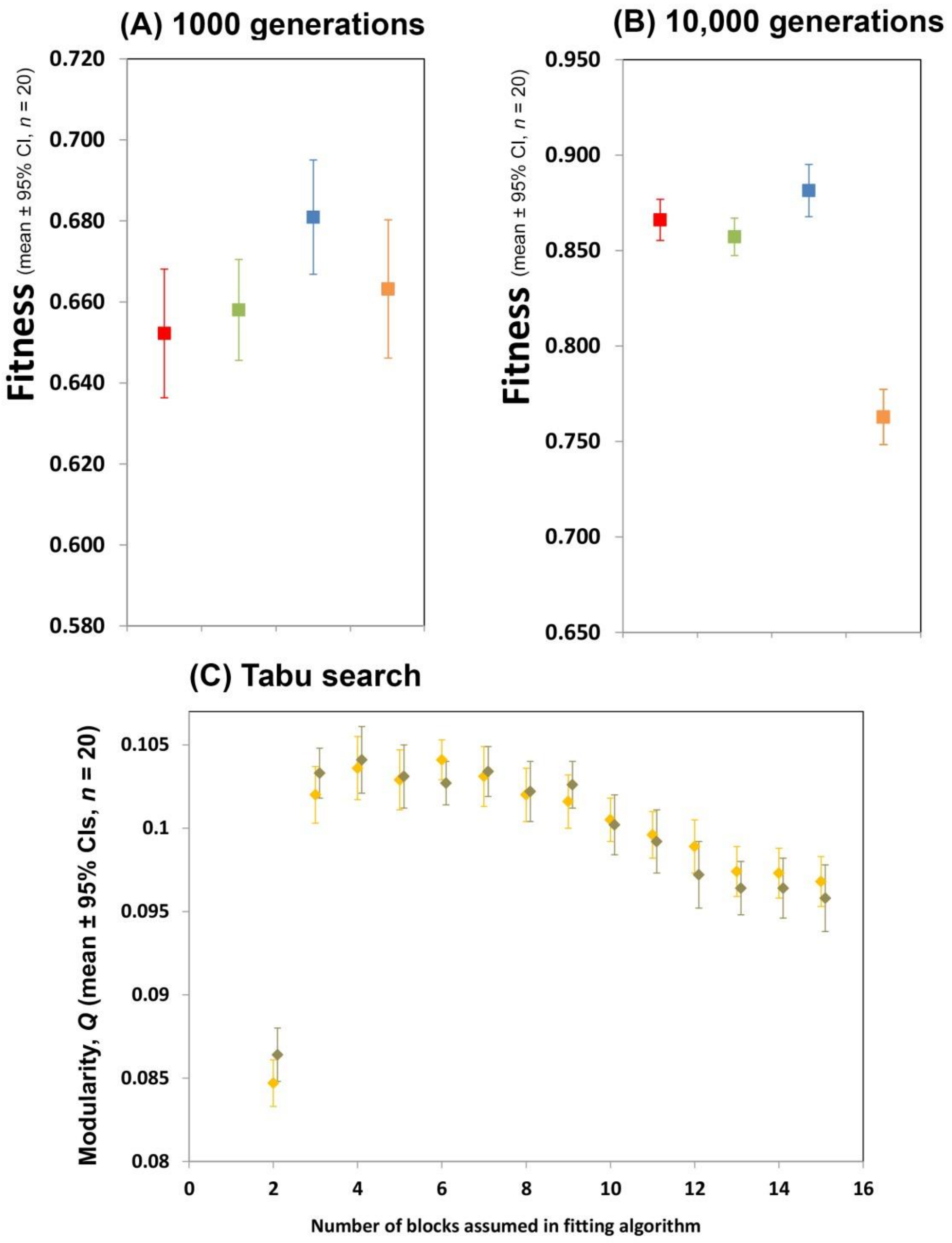

Figure S2. Evolution of modularity with reduced rate of weight mutation in the algorithm in which both networks weights and connections could evolve. Parts A and B, red, green and blue show the performance at 1000 and 10,000 generations of the fully connected non modular network (red), the sparse non-modular network (green), and the perfectly modular network (blue) of Tosh (2014), where networks were conformed as present but had a variety of fixed connective architectures and only weights were allowed to evolve. The data in yellow is the performance of the networks where both weights and connective architecture have been allowed to evolve. Part C shows the final level of evolved modularity between the input and hidden layer in the structurally evolving networks after 10,000 generations. Modularity has been assed using a Tabu search algorithm. The grey points are a control in which the degree distribution of each node in the input layer of the networks at the end of the evolutionary simulation has been randomized. No difference between grey and yellow bars means essentially that there is no more evolved modularity than would be expected at random. The number of active connections between input and hidden layer at the end of simulations was 2301 ± 14 (n = 20), approximately 0.50 of the possible number of active connections.

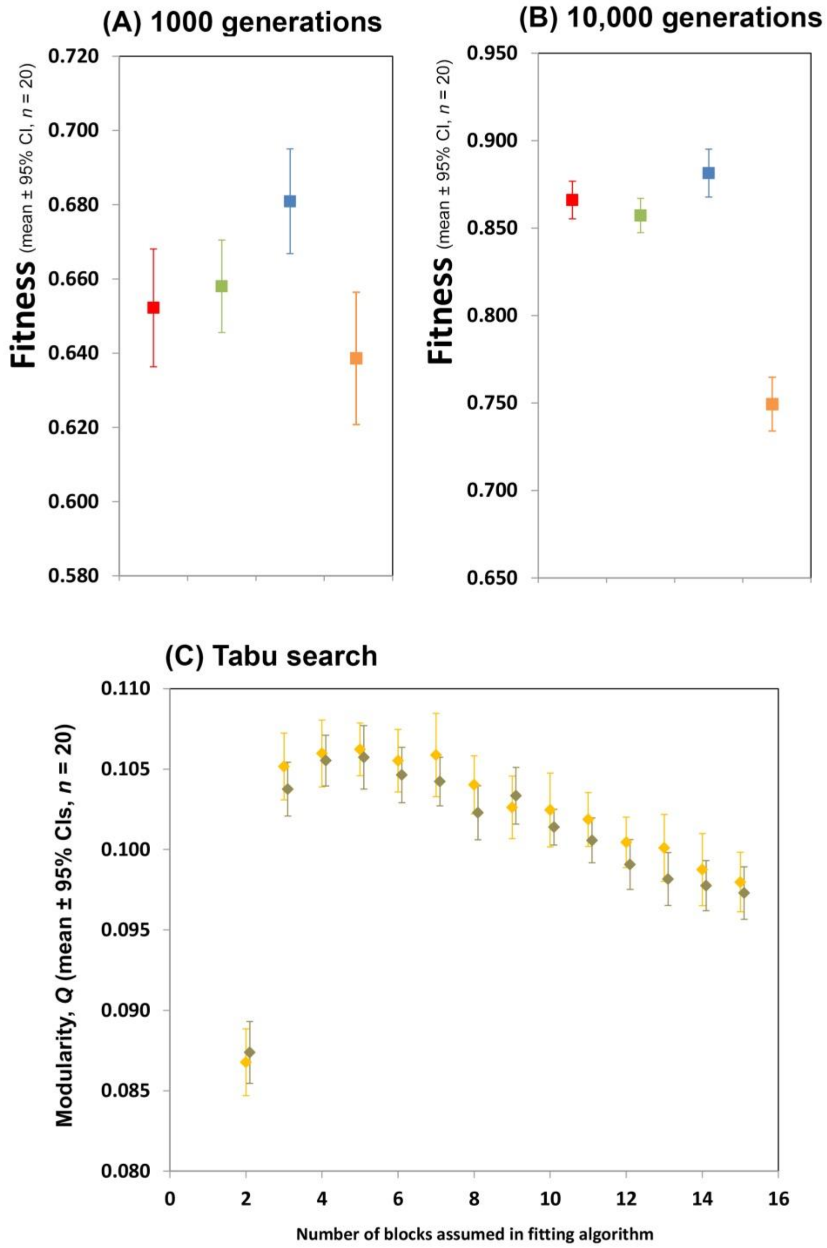

Figure S3. Evolution of modularity with reduced rate of weight mutation in the algorithm in which both networks weights and connections could evolve. Simulations additionally begin from a starting condition in which there are no connections between layers 1 and 2 of the network. Parts A and B, red, green and blue show the performance at 1000 and 10,000 generations of the fully connected non modular network (red), the sparse non-modular network (green), and the perfectly modular network (blue) of Tosh (2014), where networks were conformed as present but had a variety of fixed connective architectures and only weights were allowed to evolve. The data in yellow is the performance of the networks where both weights and connective architecture have been allowed to evolve. Part C shows the final level of evolved modularity between the input and hidden layer in the structurally evolving networks after 10,000 generations. Modularity has been assed using a Tabu search algorithm. The grey points are a control in which the degree distribution of each node in the input layer of the networks at the end of the evolutionary simulation has been randomized. No difference between grey and yellow bars means essentially that there is no more evolved modularity than would be expected at random. The number of active connections between input and hidden layer at the end of simulations was 2378 ± 230 (n = 20), approximately 0.52 of the possible number of active connections.

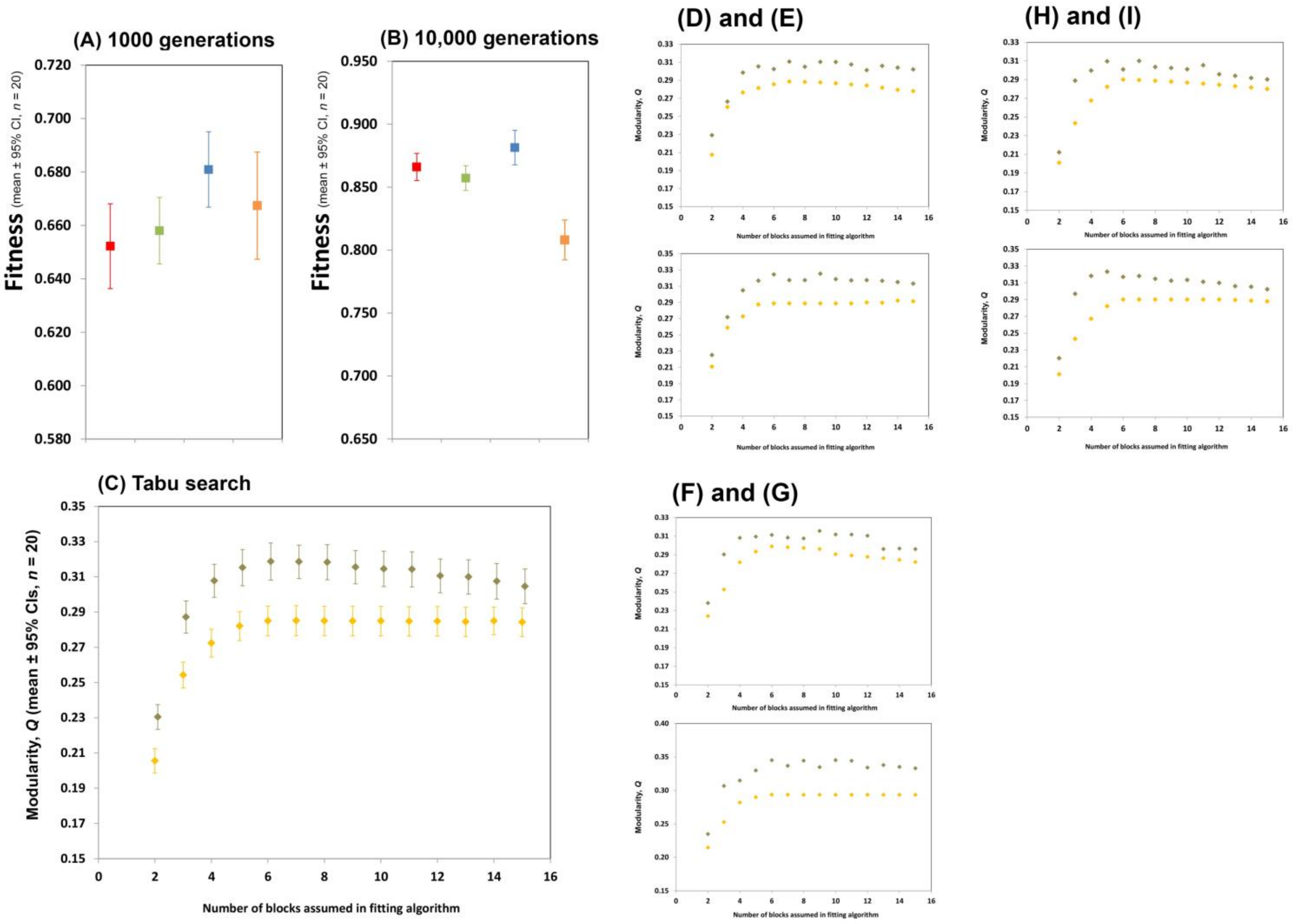

Figure S4. Evolution of modularity with reduced rate of weight mutation in the algorithm in which both networks weights and connections could evolve. Additionally, structural mutations between layers 1 and 2 of the networks are irreversible i.e. connections can only disappear. Parts A and B, red, green and blue show the performance at 1000 and 10,000 generations of the fully connected non modular network (red), the sparse non-modular network (green), and the perfectly modular network (blue) of Tosh (2014), where networks were conformed as present but had a variety of fixed connective architectures and only weights were allowed to evolve. The data in yellow is the performance of the networks where both weights and connective architecture have been allowed to evolve. Part C shows the final level of evolved modularity between the input and hidden layer in the structurally evolving networks after 10,000 generations. Modularity has been assed using a Tabu search algorithm. The grey points are a control in which the degree distribution of each node in the input layer of the networks at the end of the evolutionary simulation has been randomized. Unlike previous simulations it was found that some nodes became completely disconnected during evolution. D, H and F are the analysis of C repeated for three individual reps but with disconnected nodes removed from the network. E, G, and I are the same analyses including all nodes. The difference between selected and randomized connections in C is not entirely due to evolved node disconnection. The number of active connections between input and hidden layer at the end of simulations was 538 ± 21 (n = 20), approximately 0.12 of the possible number of active connections.

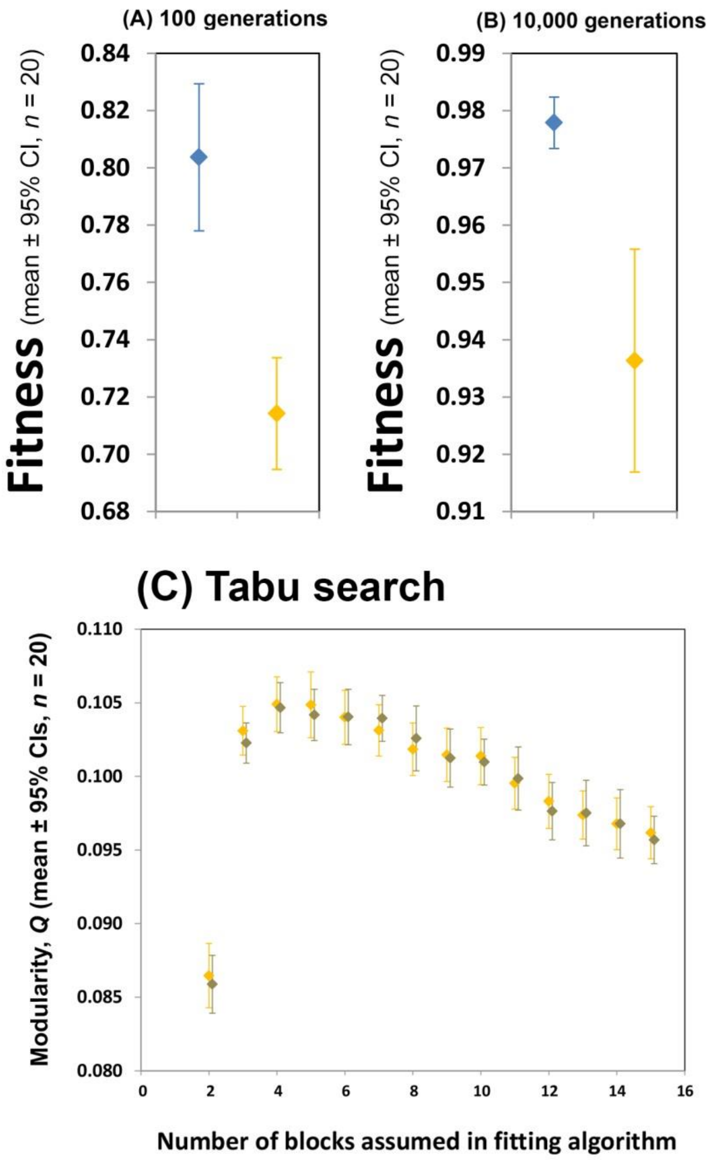

Figure S5. Evolution of modularity when both network weights and connections could evolve but the networks was given a different task to optimise. Parts A and B, red, green and blue show the performance at 100 and 10,000 generations of the perfectly modular network (blue) of Tosh (2014), where networks were conformed as present but had a variety of fixed connective architectures and only weights were allowed to evolve. The data in yellow is the performance of the networks where both weights and connective architecture have been allowed to evolve. Part C shows the final level of evolved modularity between the input and hidden layer in the structurally evolving networks after 10,000 generations. Modularity has been assed using a Tabu search algorithm. The grey points are a control in which the degree distribution of each node in the input layer of the networks at the end of the evolutionary simulation has been randomized. No difference between grey and yellow bars means essentially that there is no more evolved modularity than would be expected at random. The number of active connections between input and hidden layer at the end of simulations was 2285 ± 19 (*n* = 20), approximately 0.50 of the possible number of active connections.

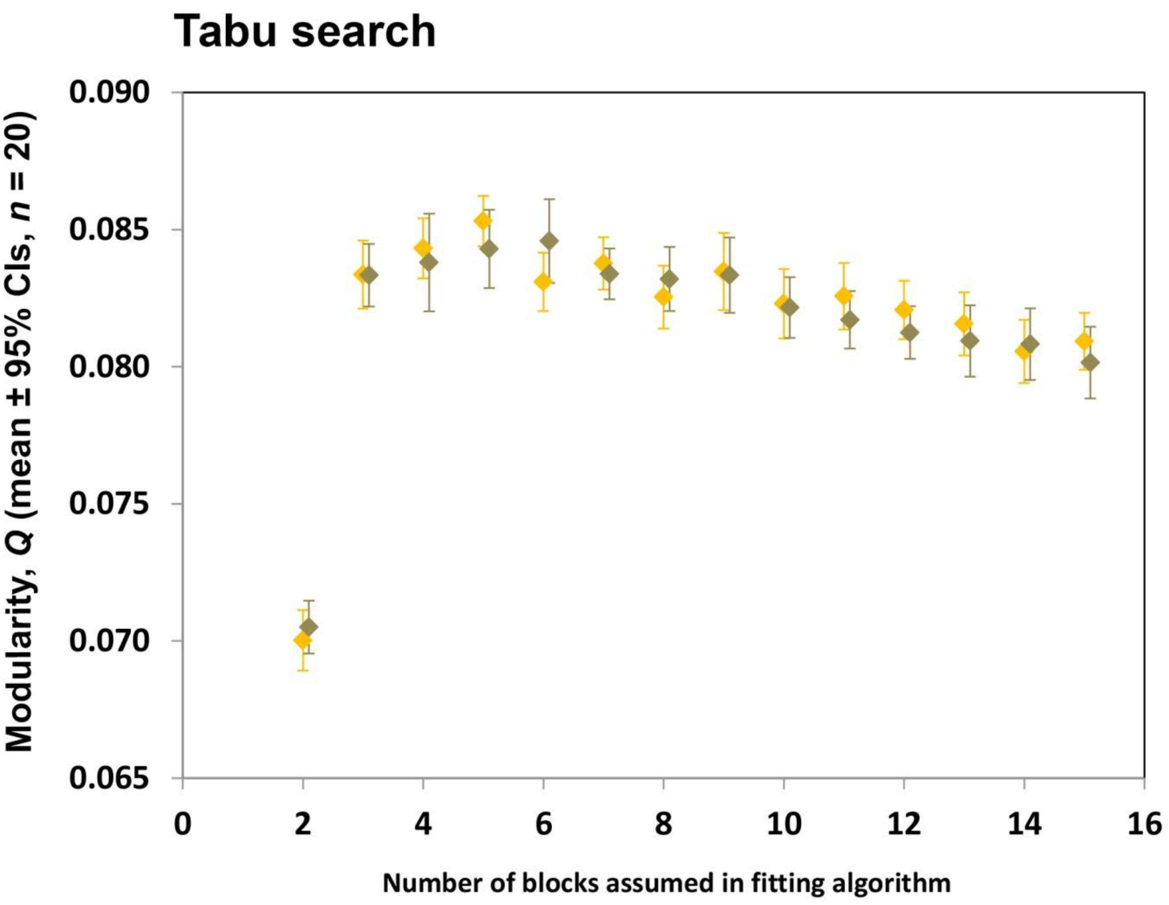

Figure S6. Evolution of modularity when both network weights and connections could evolve and the network is even larger than the starting network state (now 144/72/1 nodes in input/hidden/output layers). The plot shows how the final level of evolved modularity between the input and hidden layer in the structurally evolving networks after 10,000 generations. Modularity has been assed using a Tabu search algorithm. The data in yellow is the performance of the networks where both weights and connective architecture have been allowed to evolve. The grey points are a control in which the degree distribution of each node in the input layer of the networks at the end of the evolutionary simulation has been randomized. No difference between grey and yellow bars means essentially that there is no more evolved modularity than would be expected at random. The number of active connections between input and hidden layer at the end of simulations was 5179 ± 25 (n = 20), approximately 0.50 of the possible number of active connections.

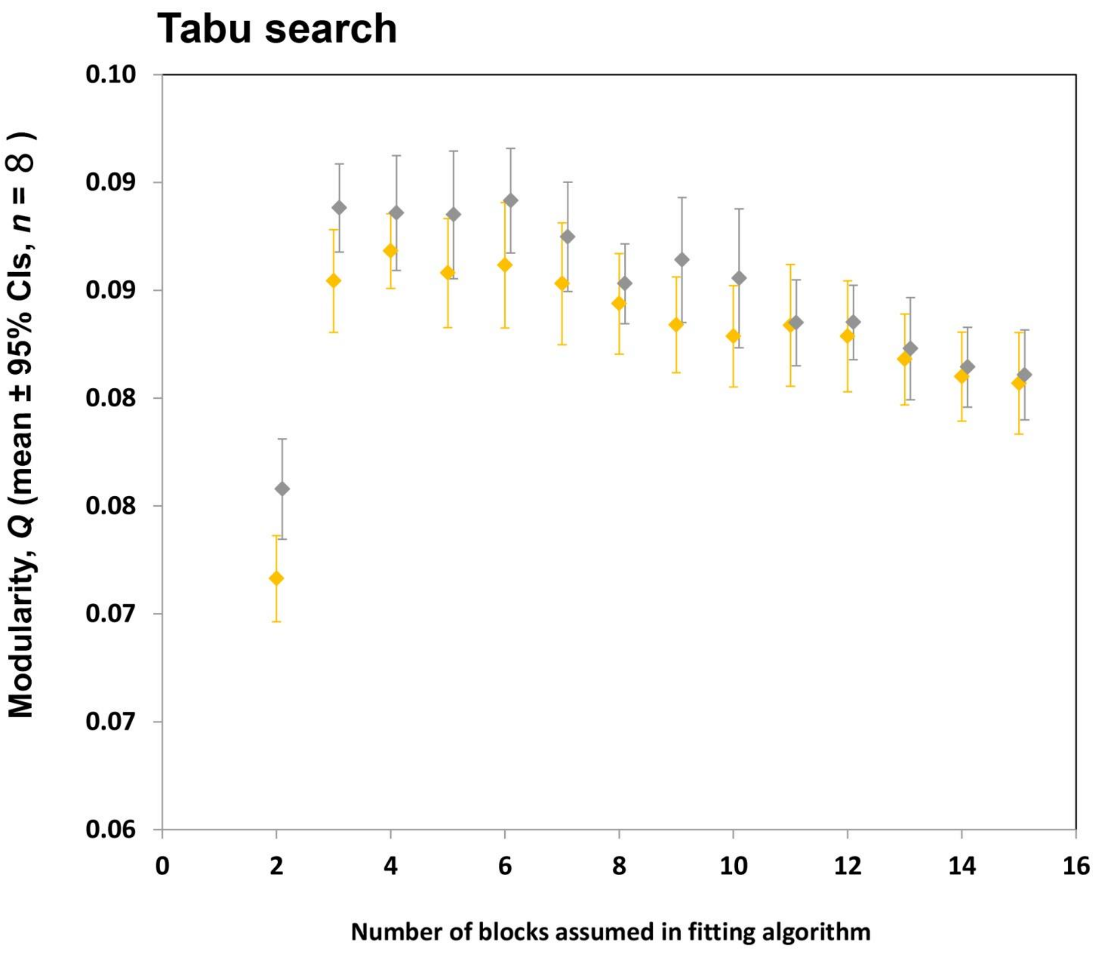

Figure S7. Evolution of modularity in an algorithm in which, in each of 400 generations, connective mutation occurs to each network in a population. Each of these mutated networks is subsequently subject to adaptive evolution for 400 generations. The whole algorithm is analogous to a scenario in which structural evolution occurs across generations of the organisms and organisms learn within each generation. The data in yellow is the final state of modularity between input and hidden layer of 8 replicate simulations subject to the regime just described. Modularity has been assed using a Tabu search algorithm. The grey points are a control in which the degree distribution of each node in the input layer of the networks at the end of the evolutionary simulation has been randomized. No difference between grey and yellow bars means essentially that there is no more evolved modularity than would be expected at random.

An analysis of selection on connective architecture

Some of the data in the sensitivity analysis presented a conundrum. The observation that networks did not realise their full performance potential when connective evolution was introduced indicates that connective architecture is not selectively neutral, but most large networks ended evolution with around half of the potential active connective possible: a scenario consistent with random drift of connections. To investigate further I repeated the simulations shown in Figure 4 of the main text and followed the number of active connections through evolutionary time. I do not know what aspects of connective architecture are being selected for but 'number of active connections' is an easily traceable measure that has the potential to be correlated to whatever aspect of connective architecture are under selection i.e. it can be used as a potential signature of selection. These simulations were repeated but allowing networks to drift by selecting networks for use in the next generation at random. Output is shown in Figure S8. Network connections do not simply drift. There is evidence for selection between generations 0 and 5000 but thereafter number of connections in networks under selection and drifting is the same. This is consistent with a scenario where most of the selection on connective architecture occurs early in simulations and thereafter connections are free to drift but other scenarios cannot be excluded. For example, selection after 5000 generations could switch to aspects of connective architecture that are not correlated with 'number of active connections'.

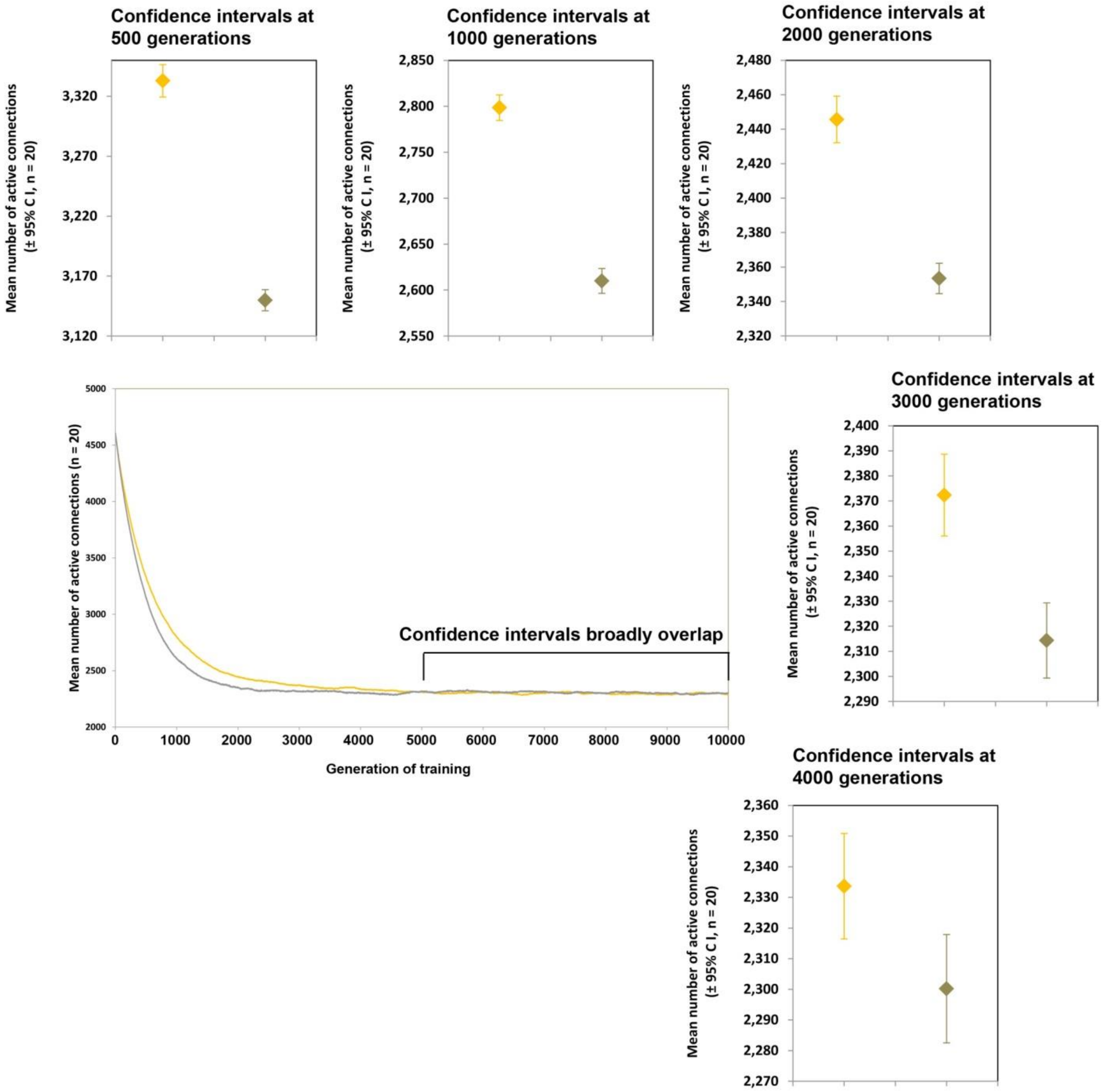

Figure S8. Active connection number vs generation of training in a repeat of the simulations of Figure 4 (Yellow). The same measure is shown in grey when the same networks are subject to random drift. Confidence intervals are too small to be shown in the main figure so are show expanded around the main figure.

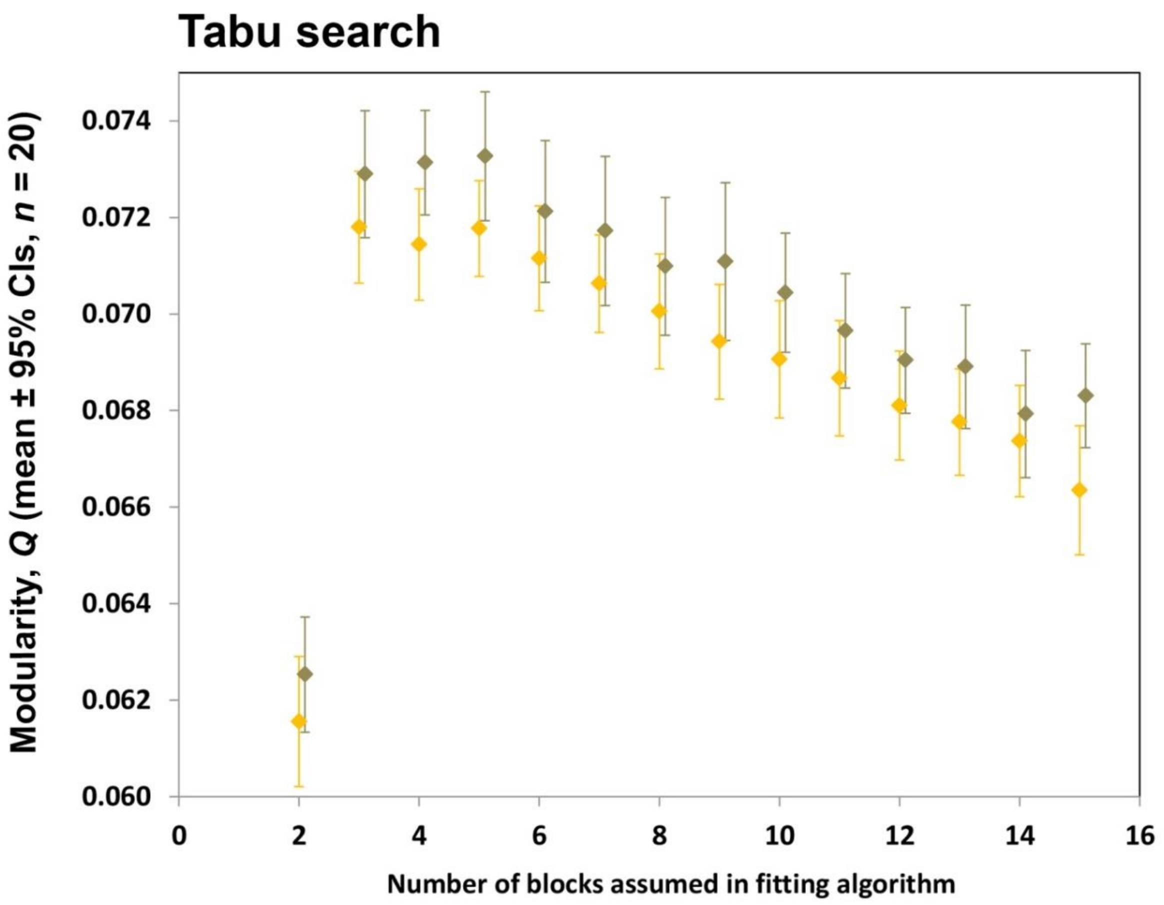

Figure S9. Simulations as Figure 4 and Figure 10 but modularity was quantified after 750 generations.

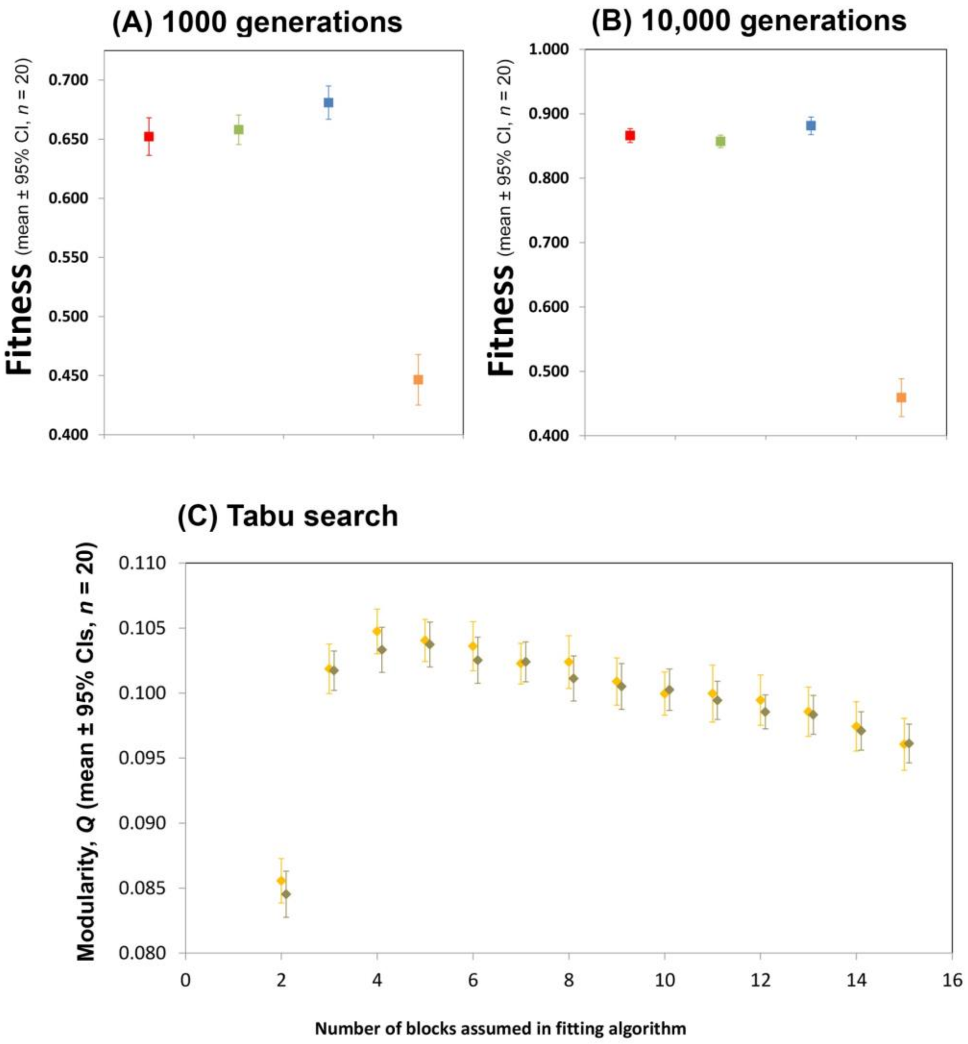

Figure S10. Evolution of modularity when both networks weights and connections could evolve. The system is parameterized as in the ‘starting network state’ of the main text with an additional increase in the rate of connective evolution from 1/1000 to 1/100. This is the most aggressive mutational regime used anywhere in the study. Parts A and B, red, green and blue show the performance at 1000 and 10,000 generations of the fully connected non modular network (red), the sparse non-modular network (green), and the perfectly modular network (blue) of Tosh (2014), where networks were conformed as present but had a variety of fixed connective architectures and only weights were allowed to evolve. The data in yellow is the performance of the networks where both weights and connective architecture have been allowed to evolve. Part C shows the final level of evolved modularity between the input and hidden layer in the structurally evolving networks after 10,000 generations. Modularity has been assed using a Tabu search algorithm. The grey points are a control in which the degree distribution of each node in the input layer of the networks at the end of the evolutionary simulation has been randomized. No difference between grey and yellow bars means essentially that there is no more evolved modularity than would be expected at random. The number of active connections between input and hidden layer at the end of simulations was 2298 ± 18 (*n* = 20), approximately 0.50 of the possible number of active connections.

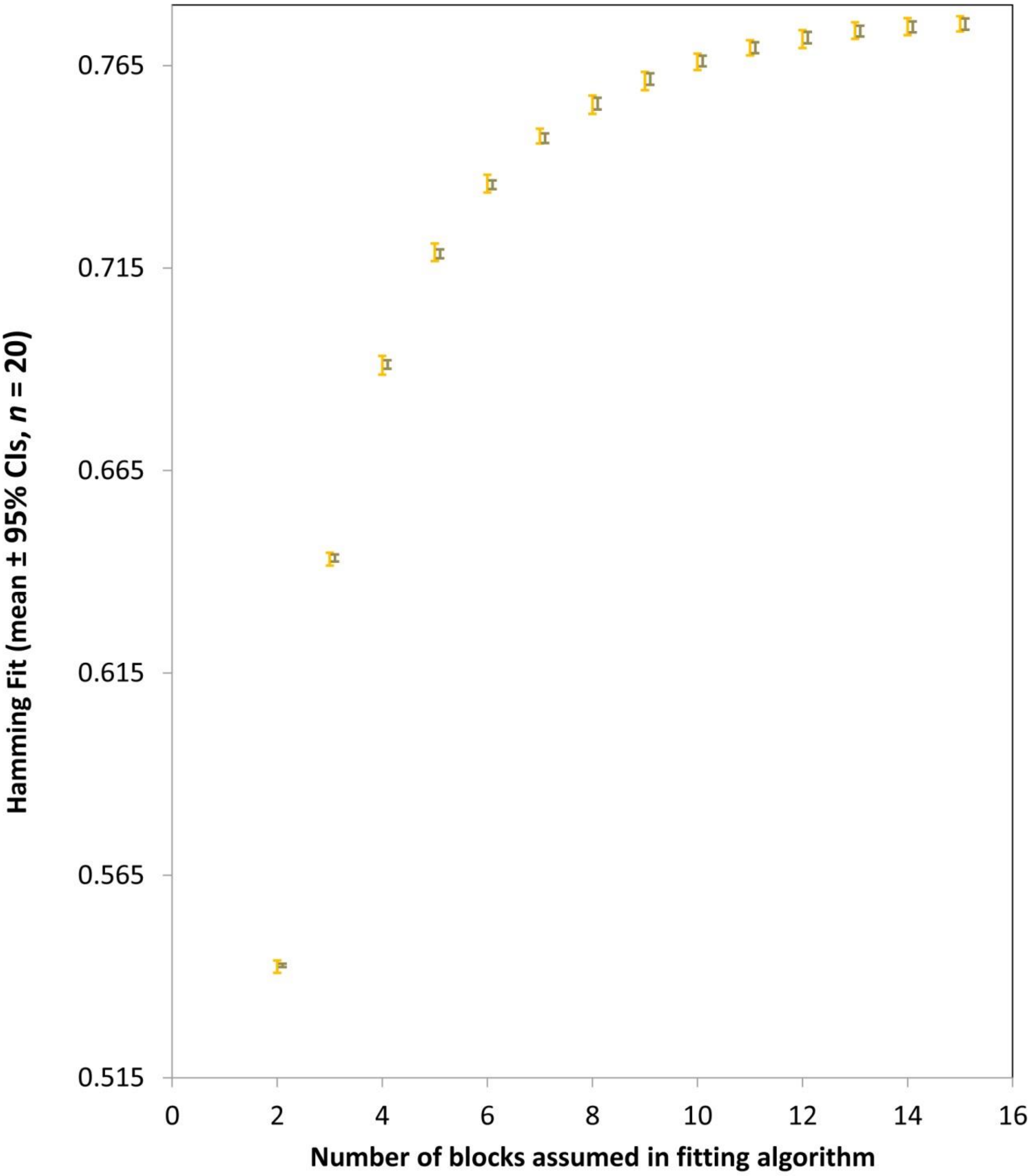

Figure S11. Evolution of modularity in the ‘starting network state’ described in the Methods section ‘*Starting network state and commonalities of all networks’*. The figure shows the final level of evolved modularity between the input and hidden layer in the structurally evolving networks after 10,000 generations (yellow). Modularity has been assessed using a Tabu search and Hamming fit. The Hamming fit counts the total number of errors: the number of zeros in a diagonal block and the number of ones in an off diagonal block. The grey points are a control in which the degree distribution of each node in the input layer of the networks at the end of the evolutionary simulation has been randomized. No difference between grey and yellow bars means essentially that there is no more evolved modularity than would be expected at random. This data complements that of Figure 2 of the main text.
